# Supplementary material for: Cerebral [18F]-FDOPA Uptake in Autism Spectrum Disorder and Its Association with Autistic Traits
Source: Diagnostics (Basel). 2021 Dec 20;11(12):2404. doi: 10.3390/diagnostics11122404 (PMC8700159; doi:10.3390/diagnostics11122404)
Supplement: Supplementary file 1 [file diagnostics-11-02404-s001.zip › diagnostics-1411384-supplementary.pdf]

Supplementary Methods 1

Participant exclusion criteria related to psychotropic medication use:

1. Use of an antipsychotic (ever) if prescribed for a psychotic disorder, as a former psychotic disorder is an exclusion criterion. If not prescribed for psychosis, then:
    a. Incidental former use of antipsychotic was allowed, if last use had been >1 year ago.
    b. Regular former use of antipsychotic was allowed, if last use had been >2 years ago.
    c. Antipsychotic formerly administered as depot medication was allowed, if last injection had been >2 years ago.
2. Use of the antipsychotic quetiapine (ever), if prescribed for a psychotic disorder. In other cases:
    a. Consumption was allowed if previously consumed in a low dose (≤50mg), but last use had been >3 months ago.
    b. Consumption was allowed if previously consumed in a high dose (>50mg), but last use had been >6 months ago.
3. Use of medication usually prescribed for attention-deficit/hyperactivity disorder (ADHD) (e.g., methylphenidate). Individuals who had stopped using these drugs for >1 year could participate.
4. Use of benzodiazepine or promethazine, unless last use had been >1 month ago.
5. Use of other psychotropic medication, unless last use had been >3 months ago.

Supplementary Methods 2

We conducted Bayesian linear regression analyses to, for each of the analysis of interest, quantify the evidence for the alternative hypothesis relative to the null-hypothesis, as expressed by the Bayes factor BF_{10} (Kelter, 2020). Default JASP priors were used (JZS prior with the r scale of 0.354).

The results in Supplementary Table 1 consistently indicate ‘anecdotal’ (BF_{10} = 1/3–1), ‘substantial’ (BF_{10} = 1/10–1/3), or ‘strong’ (BF_{10} = 1/30–1/10) evidence for the null-hypothesis (‘no association’) over the alternative hypothesis (Wagenmakers et al., 2011).

Supplementary references

Kelter, R. (2020). Bayesian alternatives to null hypothesis significance testing in biomedical research: a non-technical introduction to Bayesian inference with JASP. *BMC Medical Research Methodology*, *20*, 142.

Wagenmakers, E.-J., Wetzels, R., Borsboom, D., & van der Maas, H. L. J. (2011). Why psychologists must change the way they analyze their data: The case of psi: Comment on Bem (2011). *Journal of Personality and Social Psychology*, *100*(3), 426-432.

Table S1. *Bayes factors (BF_{10}) for the analyses examining striatal [^{18}F]-FDOPA uptake (k_i^{cer} min^{-1}) in autistic adults and controls, and its association with self-reported autistic traits.*

	k_i^{cer}	Association between k_i^{cer} value and AQ scores								
		ASD			Controls			Combined sample		
ROI	Difference ASD and controls	Total	Social	Detail	Total	Social	Detail	Total	Social	Detail
	BF_{10}	BF_{10}	BF_{10}	BF_{10}	BF_{10}	BF_{10}	BF_{10}	BF_{10}	BF_{10}	BF_{10}
Whole striatum	0.209	0.073	0.073	0.331	0.248	0.248	0.240	0.210	0.225	0.327
Putamen	0.116	0.052	0.062	0.260	0.302	0.290	0.319	0.134	0.165	0.123
Nucleus accumbens	0.147	0.054	0.048	0.496	0.303	0.305	0.277	0.131	0.160	0.136
Caudate nucleus	0.352	0.107	0.094	0.251	0.238	0.248	0.245	0.401	0.357	0.850

ASD, autism spectrum disorder; AQ, Autism Spectrum Quotient; ROI, region-of-interest; Total, AQ total scores; Social, AQ social interaction subscale scores; Detail, AQ attention to detail subscale scores; BF_{10}, Bayes factor in favor of alternative hypothesis relative to null-hypothesis.

Analyses adjusted for age, sex, smoking status, and PET/CT scanner type.
